# Health Communication Through News Media During the Early Stage of the COVID-19 Outbreak in China: Digital Topic Modeling Approach

**DOI:** 10.2196/19118

**Published:** 2020-04-28

**Authors:** Qian Liu, Zequan Zheng, Jiabin Zheng, Qiuyi Chen, Guan Liu, Sihan Chen, Bojia Chu, Hongyu Zhu, Babatunde Akinwunmi, Jian Huang, Casper J P Zhang, Wai-Kit Ming

**Affiliations:** 1 School of Journalism and Communication National Media Experimental Teaching Demonstration Center Jinan University Guangzhou, Guangdong Province China; 2 Department of Communication University at Albany State University of New York Albany, New York State, NY United States; 3 Department of Public Health and Preventive Medicine School of Medicine Jinan University Guangzhou, Guangdong Province China; 4 International School Jinan University Guangzhou, Guangdong Province China; 5 Computer Centre Jinan University Guangzhou, Guangdong Province China; 6 Center for Genomic Medicine Massachusetts General Hospital Harvard Medical School Boston, MA United States; 7 Pulmonary and Critical Care Medicine Unit, Department of Medicine Brigham and Women’s Hospital Harvard Medical School Boston, MA United States; 8 Multidisciplinary, Collaborative Research Centre for Environment and Health, Department of Epidemiology and Biostatistics School of Public Health, St Mary’s Campus Imperial College London London United Kingdom; 9 School of Public Health Li Ka Shing Faculty of Medicine The University of Hong Kong Hong Kong

**Keywords:** coronavirus, COVID-19, outbreak, health communication, mass media, public crisis, topic modeling

## Abstract

**Background:**

In December 2019, a few coronavirus disease (COVID-19) cases were first reported in Wuhan, Hubei, China. Soon after, increasing numbers of cases were detected in other parts of China, eventually leading to a disease outbreak in China. As this dreadful disease spreads rapidly, the mass media has been active in community education on COVID-19 by delivering health information about this novel coronavirus, such as its pathogenesis, spread, prevention, and containment.

**Objective:**

The aim of this study was to collect media reports on COVID-19 and investigate the patterns of media-directed health communications as well as the role of the media in this ongoing COVID-19 crisis in China.

**Methods:**

We adopted the WiseSearch database to extract related news articles about the coronavirus from major press media between January 1, 2020, and February 20, 2020. We then sorted and analyzed the data using Python software and Python package Jieba. We sought a suitable topic number with evidence of the coherence number. We operated latent Dirichlet allocation topic modeling with a suitable topic number and generated corresponding keywords and topic names. We then divided these topics into different themes by plotting them into a 2D plane via multidimensional scaling.

**Results:**

After removing duplications and irrelevant reports, our search identified 7791 relevant news reports. We listed the number of articles published per day. According to the coherence value, we chose 20 as the number of topics and generated the topics’ themes and keywords. These topics were categorized into nine main primary themes based on the topic visualization figure. The top three most popular themes were prevention and control procedures, medical treatment and research, and global or local social and economic influences, accounting for 32.57% (n=2538), 16.08% (n=1258), and 11.79% (n=919) of the collected reports, respectively.

**Conclusions:**

Topic modeling of news articles can produce useful information about the significance of mass media for early health communication. Comparing the number of articles for each day and the outbreak development, we noted that mass media news reports in China lagged behind the development of COVID-19. The major themes accounted for around half the content and tended to focus on the larger society rather than on individuals. The COVID-19 crisis has become a worldwide issue, and society has become concerned about donations and support as well as mental health among others. We recommend that future work addresses the mass media’s actual impact on readers during the COVID-19 crisis through sentiment analysis of news data.

## Introduction

In December 2019, some pneumonia cases caused by an unknown pathogen were reported in Wuhan, Hubei, China, and similar cases were soon reported in other provinces of China. After multiple sample collections and laboratory analyses, the pathogen was identified as a novel coronavirus named severe acute respiratory syndrome coronavirus 2 by the International Committee of Taxonomy of Viruses [1], and the disease was named coronavirus disease (COVID-19) by the World Health Organization on February 11, 2020 [2]. According to the National Health Commission (NHC) of the People’s Republic of China, until February 2020, there had been approximately 80,000 confirmed cases and more than 2000 deaths in China [3]. Other countries such as Japan, South Korea, Thailand, Singapore, and the United States also reported COVID-19 cases in their countries [4]. Although the cases at the early stage in these countries were identified as imported cases from Wuhan or other cities in Hubei Province, some domestic cases and local transmission were also reported.

The rapid spread of COVID-19 has already caused great public attention and many heated discussions, and the Chinese mass media have been reporting relevant information about the virus and the outbreak. As effective public health measures are required to be implemented in time to avoid the breakdown of the health system [5], the media can certainly play a crucial role in conveying updated policies and regulations from authorities to the citizens.

Since no COVID-19 vaccine is yet available, each citizen should be aware of the harm caused by this novel coronavirus, the prevention methods, and the designated hospital in their local area to access at any time. If misleading or incorrect information was transmitted to the public, the people may get anxious and react to the information in many ways, including making a panic purchase and trying unnecessary or even detrimental medicine regimens. Therefore, it requires mass media information dissemination activities in conjunction with the health stakeholders to help individuals, authorities, the government, and others to understand the precarious worldwide and public health conditions posed by COVID-19 and identify health-related knowledge and training required in facing this menace.

Given the desire to know whether the media works efficiently in delivering the latest COVID-19 information to the public audience, major media reports were collected and analyzed. Multimodal data modeling can combine multiple information reports from various resources. To cope with multimodal data, topic modeling was used. Topic modeling is a type of statistical model that arranges unstructured data structurally in accordance with latent themes. With this model, we could investigate the patterns of health communication through the media and the role the media has played so far during the COVID-19 crisis in China.

## Methods

### Data Collection

We collected Chinese news and articles related to COVID-19 from January 1, 2020, to February 20, 2020. We then applied the latent Dirichlet allocation (LDA) modeling method to derive useful information from these news reports.

Data from Chinese news and related articles were collected from the WiseSearch database [6]. The WiseSearch database is one of the most reputable, ever-growing Chinese media content databases, containing the news and article data from more than 1500 print media sources and over 10,000 internet media sources. It is famous for its reproducibility, timeliness, great coverage, and high data integrity compared with the other database [7]. The news and article data in the WiseSearch database are updated in a timely manner [6].

To gain insights into the early period of health information communication related to the coronavirus, we conducted a search with the keyword “coronavirus” in the WiseSearch database.

LDA is a generative probabilistic topic modeling method that is widely applied in text mining [8], medicine [9,10], and social network analysis [11] due to its excellent capability of converting visual words, a small part of an image that conveys a certain message about the image or alternation of the pixels, into images and visual word documents [12-14]. It is a generative statistical model with a three-level hierarchical Bayesian model. The basic assumption of this model is a combination of words belonging to different topics [15]. LDA indicates that there may be various topics in an article and that the wording in that article is attributable to one of its topics. We can discover the topics among the data pool by using Gibbs sampling techniques [16].

### Processing

A total of 11,220 articles were found with the keyword search “coronavirus,” dated between January 1, 2020, and February 20, 2020. After cleaning the data, 7791 articles remained.

**Figure 1:**
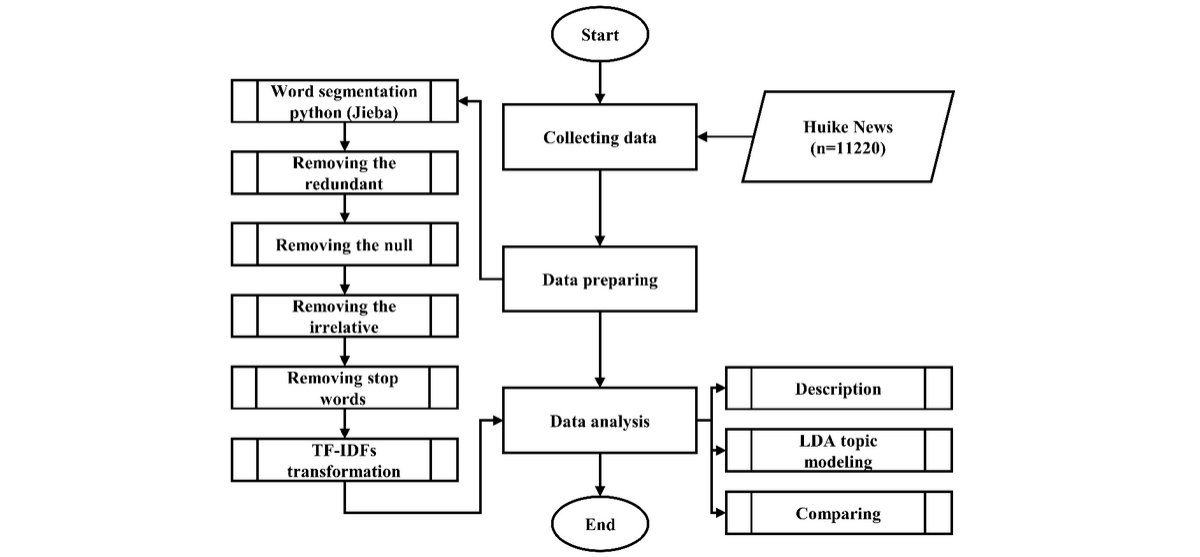
Data processing flowchart. LDA: latent Dirichlet allocation; TF–IDF: term frequency–inverse document frequency.

Before applying the LDA modeling, we used Python (Python Software Foundation) to perform data cleaning and used the Python package Jieba for data processing [17,18]. The detailed data process is illustrated in Figure 1. We next removed common Chinese stop characters such as “ten,” “a,” “of,” and “it.” We removed duplicate news reports. We then excluded news reports about other coronaviruses like severe acute respiratory syndrome-related coronavirus or Middle East respiratory syndrome-related coronavirus manually. We also built a document-term matrix and used term frequency–inverse document frequency (TF–IDF) to process the data. TF–IDF is a numerical statistic that is used to reflect the importance of a word to an article in a corpus [19].

**Figure 2:**
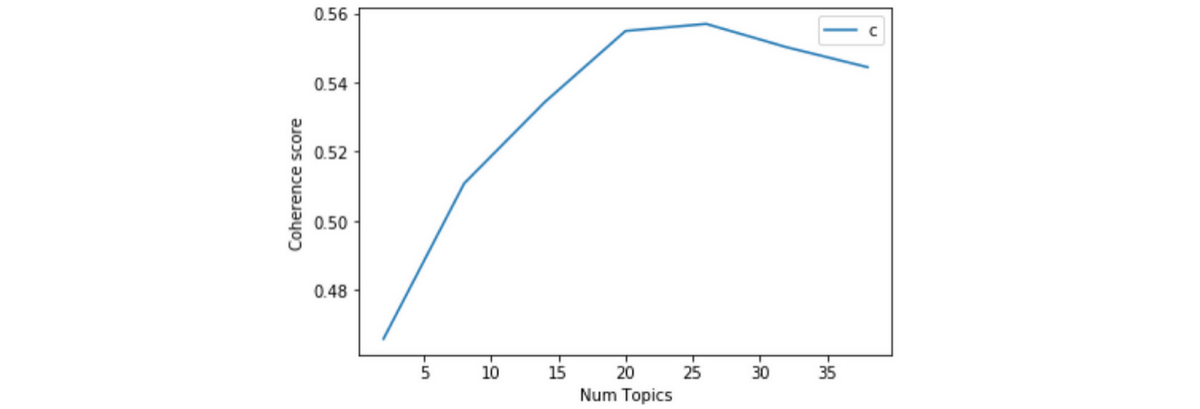
Coherence score for the topic numbers.

To seek a suitable LDA topic number and the explanations to investigate the relationship between the COVID-19 crisis and news reports, we conducted multiple studies. We used a coherence score to evaluate the selection of a suitable number of topics [20]. Topic coherence measures the consistency of a single topic by measuring the semantic similarity between words with high scores in a topic, which contributes to improving the semantic understanding of the topic. That is, words are represented as vectors by the word’s co-occurrence relation, and semantic similarity is the cosine similarity between word vectors. The coherence is the arithmetic mean of these similarities [21]. We used the Coherence Model from Gensim (RARE Technologies Ltd), the Python package for natural language processing, to calculate the coherence value [22]. According to Figure 2, the coherence score increased and reached a stable score as the number of topics increased to 20, then declined after the number of topics reached 25. However, we found that the results would be uninterpretable for humans if only statistical measures were applied [23]. As a result, we combined statistical measures and manual interpretation and chose 20 topics to analyze with the help of Python version 3.6.1 and the LDAvis tool [15]. We set λ=1 and set 20 topics and their keywords. Topics’ names were generated according to their corresponding keywords to expatiate the topics.

**Figure 3:**
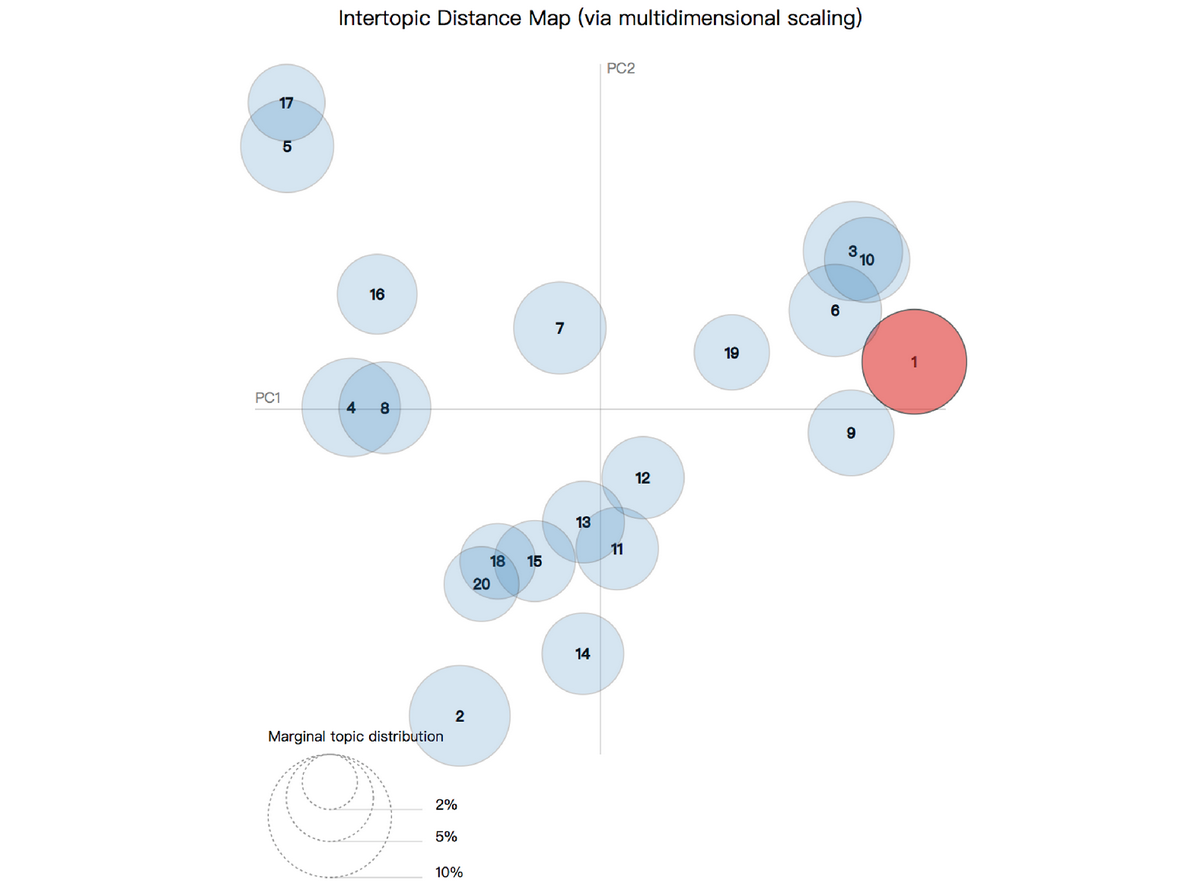
Intertopic distance map. PC: principal component.

**Figure 4:**
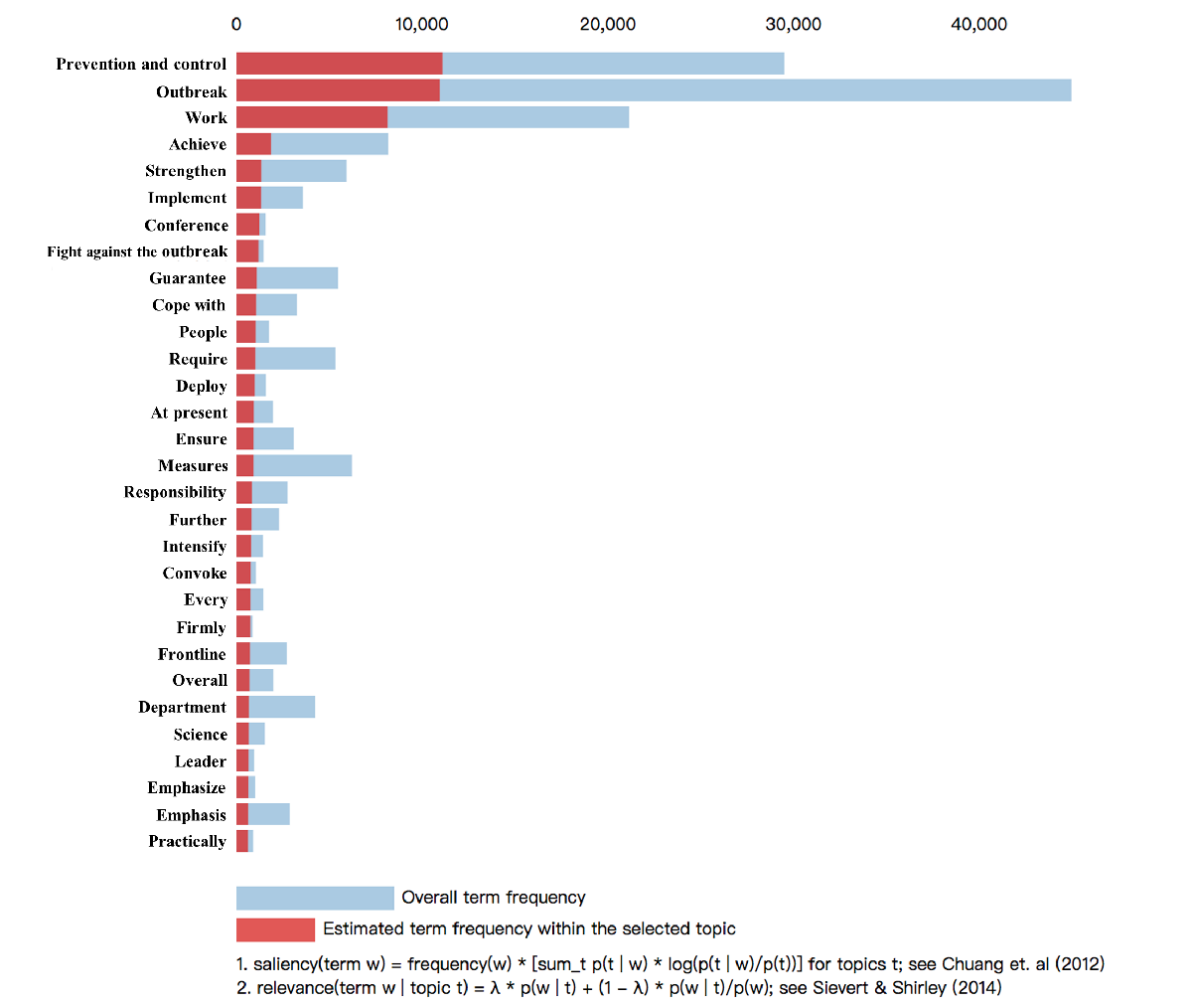
Top 30 most relevant terms for Topic 1 (7.18% of tokens).

We also divided these topics into different themes to study them better. In the visualization, that is the 2D plane (Figures 3 and 4), 20 topics were represented as cycles. These circles overlapped, and their centers are determined by computed topic distance [15]. By this approach, these 20 topics were classified into nine main primary themes and are shown in Table 1. Textbox 1 shows illustrative quotes for each theme.

**Table 1:** Topic classification and keywords.

Theme, topics, and keywords		News reports (N=7791), n (%)^a^
**Theme 1: Confirmed cases**		747 (9.58)
	Topic 5 Keywords: cases, confirmed, patient, pneumonia, novel, coronavirus, infection	444 (5.69)
	Topic 17 Keywords: new type, novel coronavirus, pneumonia, infection	303 (3.88)
**Theme 2: Medical supplies**		436 (5.59)
	Topic 7: Medical supplies Keywords: mask, disinfection, protection, contact, symptom	436 (5.59)
**Theme 3: Medical treatment and research**		1253 (16.08)
	Topic 16: Virus investigation and drug research Keywords: detection, research, laboratory, treatment, coronavirus, drug	327 (4.19)
	Topic 4: Epidemiologic study Keywords: virus, infection, spread	498 (6.39)
	Topic 8: Medical affiliation and staff Keywords: hospital, patient, medical staff, Wuhan, medical team	428 (5.49)
**Theme 4: Prevention and control procedures**		2538 (32.57)
	Topic 1: The progress in prevention and control Keywords: prevention and control, work, meeting, outbreak, fight against the outbreak, conference	560 (7.18)
	Topic 6: Community prevention and control work Keywords: personnel, prevention and control, community, outbreak, quarantine	436 (5.59)
	Topic 10: Prevention and control policy Keywords: prevention and control, work regulation, department, outbreak, measure in accordance with the law, quarantine	374 (4.80)
	Topic 3: Prevention and control measures Keywords: prevention and control, measures, outbreak	506 (6.49)
	Topic 19: Company fight against the outbreak Keywords: outbreak, company, prevention and control, coronavirus, pneumonia, impact, fight against, employee	288 (3.69)
	Topic 9: Prevention and control methods in industries and sectors Keywords: enterprise, outbreak, service, prevention and control, guarantee, support, manufacture	374 (4.80)
**Theme 5: Wuhan’s story**		522 (6.70)
	Topic 2: Wuhan’s story Keywords: Wuhan, work, Spring Festival, frontline, family member, together	
**Theme 6: Mental health**		342 (4.38)
	Topic 14: Mental health Keywords: outbreak, information, mental, society, outbreak, platform, people, nationwide, epidemic control	
**Theme 7: Global/local social/economic influences**		919 (11.79)
	Topic 20: Impact on Mainland China and Special Administrative Region of the People's Republic of China Keywords: Hong Kong, mainland, Taiwan, Macao, pneumonia, outbreak, government, impact	288 (3.69)
	Topic 18: Influence during the Spring Festival Keywords: cancel, event, hotel, visitor, Spring Festival, tourism, announce, journalist, Wuhan	296 (3.79)
	Topic 15: National and international response Keywords: China, international, response, take measures, outbreak	335 (4.29)
**Theme 8: Materials supplies and society support**		692 (8.88)
	Topic 13: Material supplies and donations Keywords: materials, donation, mask, Wuhan, antiattack, medical, prevention and control, Hubei	342 (4.38)
	Topic 11: Mask supply Keywords: mask, production, enterprise, supply, price, manufacture, market	350 (4.49)
**Theme 9: Detection at public transportation**		342 (4.38)
	Topic 12: Detection at public transportation Keywords: passenger, Wuhan, body temperature, detection, airport, vehicle	

^a^The total percentage is not 100% due to automatic rounding while exporting the results.

**Further description of each theme.**

**Theme 1**

Confirmed cases of coronavirus disease

**Theme 2**

The medical supply situation such as the shortage of surgical masks, protection suits, and safety goggles in the initial stage of the outbreak

**Theme 3**

The latest medical treatment and research about the disease, such as the designated hospital, medical staff, route of transmission, and drugs

**Theme 4**

Different aspects of the prevention and control procedures

**Theme 5**

Stories from individuals in Wuhan, such as the frontline workers combating the outbreak and lives of individuals during the crisis

**Theme 6**

The mental health of the medical staff and national citizens

**Theme 7**

Influence of coronavirus disease in China and other regions and countries, and the influence on the economy and society

**Theme 8**

The Chinese society’s cooperation to provide material support

**Theme 9**

The policy and application of detection at public transportation

## Results

Figure 3 shows the design of the topic model, in which 20 different topics are plotted as circles. The areas of the circles indicate the overall prevalence, and the center of the circles was determined by computing the distance between topics. Intertopic distances are shown on a 2D plane [24] via multidimensional scaling. The principal component (PC)1 represents the transverse axis, and the PC2 represents the longitudinal axis.

In Figure 4, we show the top 30 most relevant terms for topic 1, which had the highest proportion of all topics, as an example. We selected topic 1 and the system visualized the word frequency distribution relative to the full corpus. Each bar shows the given term’s overall frequency and the estimated frequency within topic 1. In topic 1, the news reports mainly talked about the prevention and control work deployment, and they mentioned prevention and control, outbreak work, and outbreak most frequently. In this way, we could study the content of this topic and give the topic’s name. This approach is illustrated in the literature [25].

**Figure 5:**
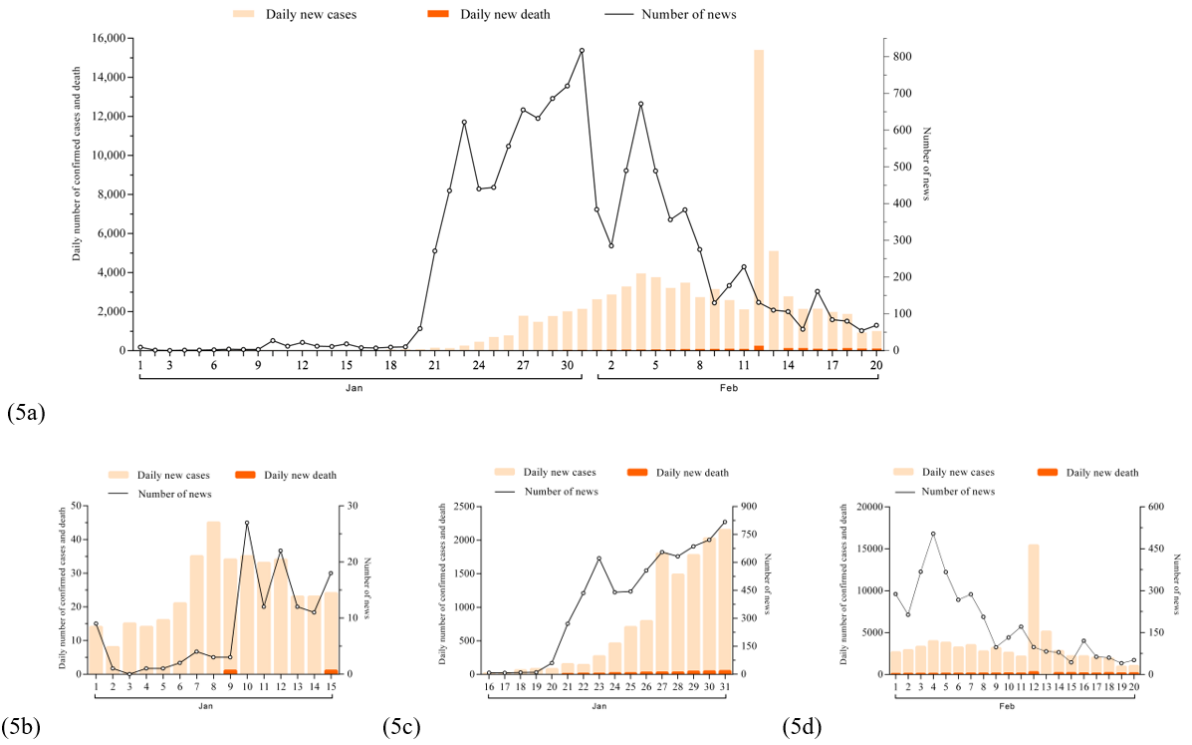
Timeseries of news streams with daily confirmed cases and deaths.

In Figure 5, 5b, 5c, and 5d are partial magnifications of 5a. The data of daily confirmed cases and deaths between January 1, 2020, and January 16, 2020, was extracted from the figure in a transmission dynamics study published on March 26, 2020 [26]. Figure 5 shows that the amount of relevant news slightly increased after a new death was reported on January 9, 2020. There was also a slight decrease between January 24 and 25, as these 2 days were Chinese New Year’s Eve and Chinese New Year, and because the Chinese government had decided to lockdown 13 cities in Hubei Province, which was accompanied by the shutdown of the transportation system on January 31, 2020. Between January 20 and 23, 2020, we observed a sharp increase in relevant news as hundreds of daily new cases occurred. We also found that there was a transient sharp decrease between January 1 and 2, 2020, after the NHC released the Protection Guideline for Population at Different Risk Levels and the Prevention Guideline of Facemask Usage [27]. As the daily new cases decreased on January 4, 2020, the number of daily news reports began to drop. The increase in the number of cases on February 12 and 13, 2020, was due to the updated diagnosis criteria in the COVID-19 protocol (fifth version) [28].

Table 1 shows the theme percentage allocation of our collected news reports. Given our analysis, theme 4 (prevention and control procedures) was the most popular theme. Theme 3 (medical treatment and research) was involved in less than one-sixth of the related news. Theme 7 (global/local social/economic influences) was included in less than one-eighth of all news reports about the coronavirus. The other 6 themes each accounted for less than 10% of the news stories.

As shown in Table 2, China News Service was the most productive media source, followed by the Securities Times and China Securities Journal. Local and national newspapers all participated in reporting recent updates.

**Table 2:** The most represented media sources for the collected news reports (N=7791).

Media sources	News reports, n (%)
China News Service	1155 (14.82)
Securities Times	176 (2.26)
China Securities Journal	159 (2.04)
Gansu Daily	121 (1.55)
Changsha Evening News	102 (1.31)
Qinghai Daily (digital newspaper)	100 (1.28)
Shenzhen Special Zone Daily	97 (1.25)
Dalian Daily (digital newspaper)	95 (1.22)
Youjiang Daily	87 (1.12)
Inner Mongolia Daily (Chinese version)	82 (1.05)

Our collected news reports mentioned various organizations and companies as shown in Table 3. Wuhan University and Huangzhong University of Science and Technology, as the top two universities in Wuhan, were the most mentioned, followed by Zhejiang University. University affinitive hospitals and university alumni associations participated actively in the fight against COVID-19.

**Table 3:** Organizations and companies mentioned in news reports (N=7791).

Organization or company	News articles, n (%)
Wuhan University	102 (1.31)
Huazhong University of Science and Technology	66 (0.85)
Zhejiang University	65 (0.83)
Pension and compensation benefits	35 (0.45)
Peking University	28 (0.36)
Wuhan Tianhe International Airport	28 (0.36)
Lanzhou University	22 (0.28)
China Construction Bank	21 (0.27)
Nanchang University	17 (0.22)
Industrial and Commercial Bank of China	17 (0.22)

## Discussion

### Principal Findings

The COVID-19 crisis has aroused great public concern in China and around the world. Topic modeling provides an alternative perspective to investigate the relationship between media reports and the COVID-19 outbreak. We collected media reports, listed the reports number each day (see Multimedia Appendix 1) and used topic modeling to analyze them. Although several COVID-19 cases were found in December 2019, we observed few news reports about them, showing that the press media did not focus on this disease at that time. As the outbreak became severe and more pneumonia cases were confirmed, the number of news reports began to steadily increase and then rapidly increased on January 19, 2020. In general, news trends peak and wane, according to the confirmed cases during other infectious disease outbreak periods; however, in some cases, the mass media cannot capture the outbreak in time and, therefore, fails to become the leading indicator [29]. This is because it takes time and rigorous effort for journalists to choose a topic, investigate the situation, collect data, and verify the authenticity of the material before they can finally present the news; as a result, a delay ensues. Mass media news reports lag behind the real time coronavirus developments, indicating that the media does not play an adequately forewarning function in public health communication and sensitization.

There was a rapid increase of related news after January 19, 2020, showing that the mass media started to pay more attention to this outbreak. However, since the virus is novel and there are not enough studies on it, the mass media might have conveyed misinformation, which may have induced negative psychological effects in the public like fear, anger, or sadness [30]. In addition, being overfed with reports will result in mass communication fatigue that will dampen the media’s effect [31]. Therefore, the government and the mass media should figure out the suitable news themes and daily news numbers to enable the public to keep alert about the outbreak with less harmful mental pressure. The media should also be obliged to ensure the reports’ accuracy.

The topics focused on by the mass media can be divided into nine classifications. Theme 4 (prevention and control procedure) and theme 3 (medical treatment and research) were two major themes that together accounted for around half of the content. It is important for the government to communicate with the citizens using mass media during the disease outbreak [32]; therefore, in these reports, the management of important government departments, medical institutions, and community control methods are emphasized. Positive and enthusiastic forecasts backed with active public health interventions are disseminated, which can eliminate unnecessary public worry and extreme panic, aimed at asserting the nation’s confidence in virus containment and victory within a short period.

Control of the sources of infection, interruptions of transmission routes, and the protection of susceptible people are three major principles to prevent and control infectious diseases. To cope with the COVID-19 crisis, the Chinese government took measures based on these three principles. The detection of viral infections within public transportation networks aroused great public concern, given that the outbreak coincided with the Spring Festival when many were traveling. Few news reports about this are included in theme 4 (prevention and control procedure), suggesting that the mass media might not have been providing sufficient health information about detection within the transportation network.

The scale of medical treatments and research was the second most popular topic. Our results showed that the mass media conveyed this kind of health information by paying attention to the detection of suspicious cases, drugs that might cure patients, and the transmission routes of the virus. However, reports within theme 4 (prevention and control procedure) and theme 3 (medical treatment and research) mainly focused on the whole society, while instructions on personal prevention and clinic and medicine choices were less mentioned.

Influences on activities (home and abroad) were also reported together with economic influences, which was included in theme 7 (global/local social/economic influences). These data indicate that the impact of the COVID-19 crisis is not limited to the medical field but also extends to social and economic fields. It is also a worldwide health issue that requires people around the world to work closely together.

The term “confirmed cases” appeared in 9.58% (n=747/7791) of the articles. This indicates that the mass media has served a public health function, as case numbers and their changing rates in news reports can directly give the public intuitive feelings about the speed of viral spread, the momentum, and the hazard of this coronavirus. It can also help citizens remain alert about virus transmission and, therefore, change their daily habits accordingly.

Theme 2 (medical supplies) and theme 8 (material supplies and society support) connect the material supplies with the COVID-19 crisis. Since the outbreak was so sudden and the transmission is so rapid, people in affected areas require medical material and other necessities, especially after the Chinese government shut the major entrance to Hubei to control the outbreak. The mass media can communicate with other parts of China to call for donations and support.

There was an emphasis on Wuhan stories, where news stories focused on the lives of individuals instead of the whole city. We also observed that theme 6 (mental health) accounts for 4.38% (342/7791) of all news articles. Previous studies have shown that there was an increase in mental health problems in both the medical staff [33] and the residents under quarantine [34] during other previous disease outbreaks. Therefore, these kinds of news reports can help the readers refocus on this easily neglected area, and therefore, early interventions can be made. These two themes indicate that the mass media adopts a people-oriented principle when reporting on the COVID-19 crisis, contributing to the warm society phenomenon.

### Limitations

This study is the first step to understanding the Chinese mass media’s role during the COVID-19 crisis. However, there are still several limitations in our study. First, we included a large number of Chinese news articles about COVID-19 from the WiseSearch mass media database, which only covers text news articles. However, the mass media has recently used new media platforms such as TikTok (video social media) and WeChat (the largest Chinese instant messaging app) to deliver health information through images, snapshots, and short videos. Therefore, we may have omitted news content and the impact of mass media in these media platforms. Second, we only selected a certain period of the outbreak. The pandemic is still ongoing, and the topics and themes are changing; therefore, we may have missed some novel topics and themes. Third, the LDA model has its own limitations such as a lack of nuances for qualitative thematic analysis and poor performance on short articles. Some relative studies introduced sentiment analysis to investigate the emotional differences in the message content [35]; it would be valuable if we could also apply sentiment analysis to supervise the news and investigate the public’s reaction to news related to COVID-19.

### Conclusion

Collecting and analyzing reports on the novel coronavirus shed light on how the Chinese media have delivered health information during the COVID-19 crisis. Our study provides evidence that the Chinese mass media news lags behind when reporting the major developments of the viral spread. Prevention and control procedures, medical treatment, and research are major themes of the press but mainly focus on the whole society, while instructions on personal and individual prevention, clinic and medicine choices, and detection need to be further enhanced. Global and local influences were reported as the COVID-19 crisis started to impose pressure on public health worldwide and urged cooperation among all humankind. Further research should be considered to explore the impacts of mass media on the readers through sentiment analysis of news data and the influences of misinformation about COVID-19 delivered through the mass media.

## Appendix

Multimedia Appendix 1 Timeseries news streams.

## Abbreviations

COVID-19: coronavirus disease
LDA: latent Dirichlet allocation
NHC: National Health Commission
PC: principal component
TF–IDF: term frequency–inverse document frequency
