# Supplementary material for: Health Communication Through News Media During the Early Stage of the COVID-19 Outbreak in China: Digital Topic Modeling Approach
Source: J Med Internet Res. 2020 Apr 28;22(4):e19118. doi: 10.2196/19118 (PMC7189789; doi:10.2196/19118)
Supplement: Multimedia Appendix 1 [file jmir_v22i4e19118_app1.docx]

| **Date** | **Article Number** |
| --- | --- |
| 2020-01-01 | 9 |
| 2020-01-02 | 1 |
| 2020-01-03 | 0 |
| 2020-01-04 | 1 |
| 2020-01-05 | 1 |
| 2020-01-06 | 2 |
| 2020-01-07 | 4 |
| 2020-01-08 | 3 |
| 2020-01-09 | 3 |
| 2020-01-10 | 27 |
| 2020-01-11 | 12 |
| 2020-01-12 | 22 |
| 2020-01-13 | 12 |
| 2020-01-14 | 11 |
| 2020-01-15 | 18 |
| 2020-01-16 | 8 |
| 2020-01-17 | 7 |
| 2020-01-18 | 9 |
| 2020-01-19 | 10 |
| 2020-01-20 | 60 |
| 2020-01-21 | 271 |
| 2020-01-22 | 435 |
| 2020-01-23 | 622 |
| 2020-01-24 | 440 |
| 2020-01-25 | 444 |
| 2020-01-26 | 556 |
| 2020-01-27 | 655 |
| 2020-01-28 | 632 |
| 2020-01-29 | 686 |
| 2020-01-30 | 720 |
| 2020-01-31 | 817 |
| 2020-02-01 | 384 |
| 2020-02-02 | 285 |
| 2020-02-03 | 490 |
| 2020-02-04 | 672 |
| 2020-02-05 | 489 |
| 2020-02-06 | 356 |
| 2020-02-07 | 383 |
| 2020-02-08 | 275 |
| 2020-02-09 | 130 |
| 2020-02-10 | 177 |
| 2020-02-11 | 228 |
| 2020-02-12 | 131 |
| 2020-02-13 | 110 |
| 2020-02-14 | 106 |
| 2020-02-15 | 58 |
| 2020-02-16 | 161 |
| 2020-02-17 | 84 |
| 2020-02-18 | 80 |
| 2020-02-19 | 54 |
| 2020-02-20 | 69 |

**Supplementary table: Time series news streams**
